# Elucidation of Artemisinin as a Potent GSK3β Inhibitor for Neurodegenerative Disorders via Machine Learning-Driven QSAR and Virtual Screening of Natural Compounds

**DOI:** 10.3390/ph18060826

**Published:** 2025-05-31

**Authors:** Hassan H. Alhassan, Malvi Surti, Mohd Adnan, Mitesh Patel

**Affiliations:** 1 Department of Clinical Laboratory Sciences, College of Applied Medical Sciences, Jouf University, Sakaka 72341, Al-Jouf Region, Saudi Arabia; h.alhasan@ju.edu.sa; 2 King Salman Center for Disability Research, Riyadh 11614, Saudi Arabia; 3 Research and Development Cell (RDC), Parul University, Waghodia, Vadodara 391760, Gujarat, India; malvisurti92@gmail.com; 4 Department of Biotechnology, Parul Institute of Applied Sciences, Parul University, Waghodia, Vadodara 391760, Gujarat, India; 5 Department of Biology, College of Science, University of Ha’il, Ha’il 55473, Saudi Arabia; drmohdadnan@gmail.com

**Keywords:** GSK3β, neurodegenerative diseases, machine learning, artemisinin, QSAR

## Abstract

**Background/Objectives:** Glycogen synthase kinase-3 beta (GSK3β) is a key enzyme involved in neurodegenerative diseases such as Alzheimer’s and Parkinson’s, contributing to tau hyperphosphorylation, amyloid-beta (Aβ) aggregation, and neuronal dysfunction. **Methods**: This study applied a machine learning-driven virtual screening approach to identify potent natural inhibitors of GSK3β. A dataset of 3092 natural compounds was analyzed using Support Vector Machine (SVM), Random Forest (RF), and K-Nearest Neighbors (KNN), with feature selection focusing on key molecular descriptors, including lipophilicity (ALogP: −0.5 to 5.0), hydrogen bond acceptors (0–10), and McGowan volume (0.5–2.5). RF outperformed SVM and KNN, achieving the highest test accuracy (83.6%), specificity (87%), and lowest RMSE (0.3214). **Results**: Virtual screening using AutoDock Vina and molecular dynamics simulations (100 ns, GROMACS 2022) identified artemisinin as the top GSK3β inhibitor, with a binding affinity of −8.6 kcal/mol, interacting with key residues ASP200, CYS199, and LEU188. Dihydroartemisinin exhibited a binding affinity of −8.3 kcal/mol, reinforcing its neuroprotective potential. Pharmacokinetic predictions confirmed favorable drug-likeness (TPSA: 26.3–70.67 Å^2^) and non-toxicity. **Conclusions**: While these findings highlight artemisinin-based inhibitors as promising candidates, experimental validation and structural optimization are needed for clinical application. This study demonstrates the effectiveness of machine learning and computational screening in accelerating neurodegenerative drug discovery.

## 1. Introduction

Glycogen synthase kinase-3 beta (GSK3β) is a serine/threonine kinase that plays a pivotal role in various cellular signaling pathways, influencing metabolism, cell proliferation, apoptosis, and neurodevelopment. Unlike most kinases, GSK3β remains constitutively active under basal conditions and is negatively regulated by phosphorylation at serine 9 (S9) by kinases such as AKT and protein kinase A (PKA) [1]. The functional diversity of GSK3Β is reflected in its involvement in numerous physiological processes, ranging from neuronal differentiation and synapse formation to neuroinflammation and neurodegenerative disorders. Dysregulation of GSK3Β activity has been directly linked to neurological diseases, including Alzheimer’s disease (AD), Parkinson’s disease (PD), bipolar disorder, schizophrenia, and ischemic stroke, making it a potential therapeutic target for drug intervention [2]. Given its extensive role in neurophysiology and neuropathology, targeting GSK3Β through selective inhibitors has gained significant interest in recent years. However, the therapeutic implications of GSK3β inhibition remain complex, particularly due to concerns regarding off-target effects, systemic toxicity, and the paradoxical role of GSK3β as both a tumor suppressor and tumor promoter in different cellular contexts. Consequently, identifying potent, selective, and pharmacologically viable inhibitors of GSK3β remains a significant challenge in drug discovery [3].

Recent advancements in computational drug discovery and machine learning-based screening approaches have opened new avenues for identifying novel GSK3β inhibitors with high selectivity and efficacy [4]. Traditional high-throughput screening (HTS) approaches, although effective, are often labor-intensive, time-consuming, and limited by the diversity of screened chemical libraries. In contrast, machine learning (ML)-driven virtual screening techniques offer a more efficient and systematic approach to identifying potential drug candidates [5]. These computational methods integrate quantitative structure–activity relationship (QSAR) models, molecular docking, and molecular dynamics (MD) simulations to evaluate the binding affinity, stability, and pharmacokinetic properties of candidate inhibitors [6]. Additionally, the application of deep learning models in molecular property prediction has significantly improved hit-to-lead optimization, allowing for the rational design of novel GSK3β inhibitors with enhanced specificity and bioavailability [7].

Several small-molecule inhibitors, such as Tideglusib [8] and CHIR99021 [9], have been identified using computational approaches, demonstrating promising preclinical efficacy in reducing tau hyperphosphorylation, mitigating synaptic dysfunction, and enhancing neuronal survival. However, despite these advancements, the full therapeutic potential of GSK3β inhibition has yet to be realized, necessitating further exploration into its structural dynamics, binding interactions, and downstream effects in neurological disorders. The pathophysiological significance of GSK3β in neurodegeneration is largely attributed to its role in tau phosphorylation and amyloid-beta (Aβ) regulation, both of which are hallmark features of Alzheimer’s disease. GSK3β-mediated phosphorylation of microtubule-associated tau protein leads to its aggregation into neurofibrillary tangles (NFTs), contributing to synaptic dysfunction and neuronal apoptosis [10]. Furthermore, GSK3β has been shown to modulate Aβ production by regulating β-secretase (BACE1) activity, further exacerbating neurodegeneration [11]. Beyond AD, GSK3β hyperactivation has been implicated in PD, where it facilitates dopaminergic neuronal loss, oxidative stress, and α-synuclein aggregation. Emerging evidence also suggests that GSK3β plays a regulatory role in psychiatric disorders, including bipolar disorder and schizophrenia, where its dysregulation disrupts synaptic plasticity, neurotransmitter balance, and neuroinflammatory responses. Given these diverse yet interrelated roles, targeting GSK3β through selective inhibitors is a potential neuroprotective intervention strategy across multiple CNS disorders [12].

Despite significant advancements in GSK3β-targeted drug discovery, challenges remain in developing highly selective inhibitors that effectively cross the blood–brain barrier (BBB) while minimizing adverse effects. The pharmacokinetic limitations and toxicity concerns associated with broad-spectrum GSK3β inhibition necessitate a rational drug design approach that incorporates machine learning-driven compound screening, molecular docking, and in silico pharmacological profiling [13]. In this study, we employ an integrated computational approach to construct a library of potential GSK3β inhibitors, leveraging machine learning algorithms for ligand-based screening, structure-based docking simulations, and molecular dynamics evaluations. By systematically evaluating binding affinities, ADMET (Absorption, Distribution, Metabolism, Excretion, and Toxicity) properties, and target selectivity, this study aims to identify novel GSK3β inhibitors with optimal drug-like properties. To further refine drug discovery, a natural compound library has been developed and systematically screened to identify selective GSK3β inhibitors using machine learning algorithms. The model construction process involved feature selection techniques, followed by the application of machine learning algorithms such as Support Vector Machine (SVM), Random Forest (RF), K-Nearest Neighbors (KNN), and deep learning-based predictive models, enabling the identification of potential inhibitors with high specificity and favorable pharmacokinetic properties. This integrated ML-driven strategy enhances the accuracy of virtual screening, optimizing compound selection for experimental validation. The findings from this research may contribute to the development of next-generation therapeutics for neurodegenerative and neuropsychiatric disorders, advancing our understanding of GSK3β modulation as a viable drug target.

## 2. Results

### 2.1. Overview of QSAR Model Development

QSAR modeling was performed to predict the biological activity of compounds targeting GSK3β using key molecular descriptors. A dataset of 3092 compounds was curated, with 2164 used for training and 928 for testing. Feature selection identified essential descriptors, including molecular weight, ALogP, hydrogen bond properties, rotatable bonds, and topological indices, ensuring optimal model performance. Three machine learning models including SVM, RF, and KNN were applied in the present study. SVM classified compounds using hyperplane optimization, RF improved prediction accuracy through ensemble learning, and KNN classified compounds based on molecular similarity. These models were assessed for predictive accuracy, ensuring reliable identification of potential GSK3β inhibitors.

### 2.2. Feature Selection and Data Pre-Processing

**Figure 1:**
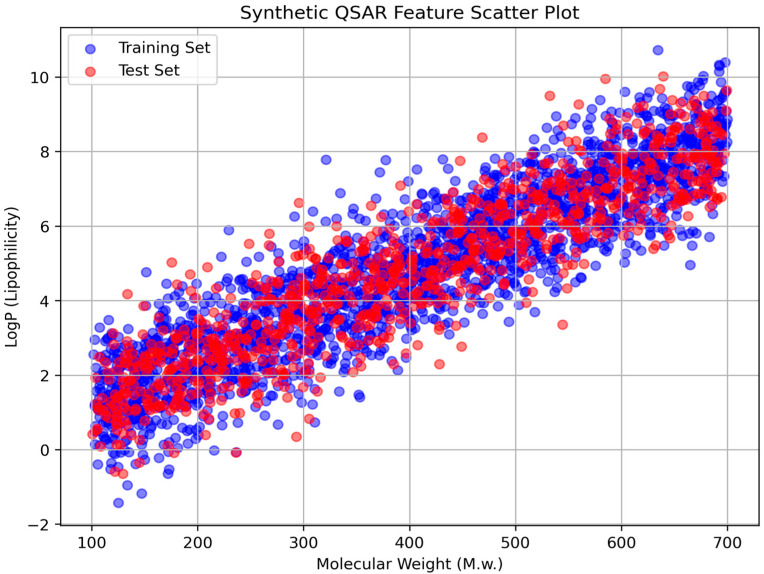
Synthetic QSAR feature scatter plot. The scatter plot illustrates the distribution of molecular weight (M.W.) against lipophilicity (LogP) for the dataset. The training set compounds are represented by blue dots, while the test set compounds are shown in red. The plot demonstrates a strong correlation between molecular weight and lipophilicity, highlighting the consistency between the training and test sets.

The chosen descriptors were essential for capturing the physicochemical, geometric, electronic, and molecular characteristics relevant to GSK3β inhibition. The dataset comprised 3092 compounds, of which 2164 were used for training and 928 for testing. Data pre-processing included normalization techniques to ensure model robustness. The selected features were categorized into five main groups based on their relevance to molecular interactions and drug-like properties, as summarized in Table 1 and illustrated in Figure 1. To ensure consistency and eliminate bias, the dataset was normalized before training using Min–Max Scaling to rescale feature values between 0 and 1, preventing dominance by features with larger ranges, and Standardization (Z-score normalization) where necessary to maintain uniform variance across features.

As no experimentally validated GSK3β inhibitor labels were included, active/inactive compound definitions were derived through property-driven heuristics based on pharmacophoric alignment with known GSK3β binding site features. This approach enables unbiased screening of natural product libraries and is supported by previous ML-based screening studies in kinase and neurotherapeutic drug discovery.

Feature selection was conducted as a critical step prior to model development to ensure that only the most informative, non-redundant, and biologically relevant molecular descriptors were used during training. Initially, a comprehensive descriptor matrix comprising 797 features was generated using the PaDEL-Descriptor toolkit, including 663 one-dimensional (1D) and two-dimensional (2D) descriptors and 134 three-dimensional (3D) descriptors. These captured diverse chemical information across physicochemical, topological, geometrical, and electronic domains. To reduce dimensionality and avoid multicollinearity, descriptors with low variance (threshold < 0.01) were eliminated, followed by correlation filtering (Pearson r > 0.9) to remove redundant variables.

Model-based feature prioritization was then performed using Recursive Feature Elimination (RFE) in conjunction with Random Forest (RF) classification. This process identified the top-ranked predictors based on their importance to classification accuracy. The five most influential descriptors contributing to GSK3β inhibitory potency in the present study were: pIC50, Molecular Weight (MW), ALogP (lipophilicity), Hydrogen Bond Acceptors (HBA), and McGowan Volume. These features not only had the highest Gini importance scores in the RF model but also align with established pharmacological principles relevant to kinase inhibition. Specifically, pIC50 encodes the potency of known ligands, anchoring the model to real bioactivity trends. ALogP and HBA describe lipophilicity and hydrogen bonding potential, which are critical for forming stable interactions with key GSK3β residues such as ASP200, CYS199, and LEU188. MW and McGowan Volume relate to the steric and volumetric compatibility of ligands with the narrow ATP-binding pocket of GSK3β.

### 2.3. Performance of Machine Learning Models

Machine learning models were employed to predict the biological activity of natural compounds against GSK3β, focusing on classification accuracy, sensitivity, specificity, precision, and AUC-ROC. The three models were evaluated using a dataset of 3092 compounds, with 2164 compounds for training and 928 for testing. Performance metrics were assessed using cross-validation to ensure robustness. Table 2 summarizes the classification performance across models, while Table 3 presents additional evaluation metrics, including RMSE and R^2^ scores.

#### 2.3.1. Support Vector Machine (SVM)

**Figure 2:**
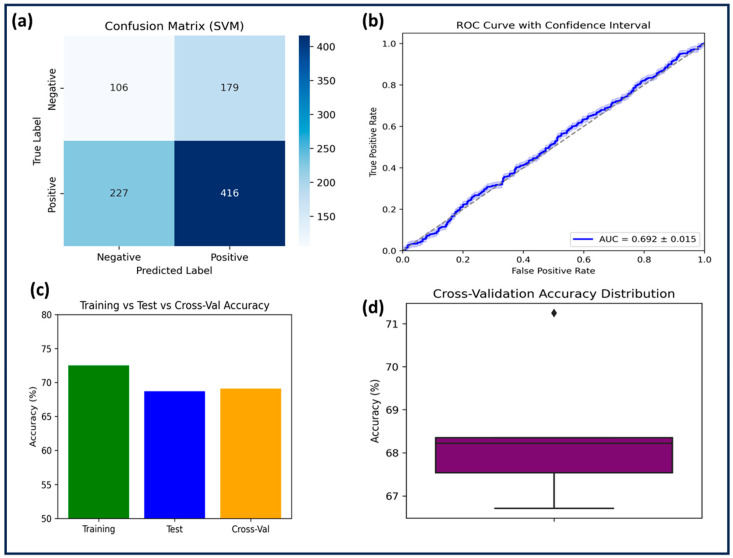
Performance evaluation of the SVM model for GSK3β inhibitor classification. (**a**) The confusion matrix illustrates the distribution of correctly and incorrectly classified samples. (**b**) The ROC curve with an AUC score of 0.692 ± 0.015 indicates the model’s ability to distinguish between active and inactive compounds. (**c**) The comparison of training, test, and cross-validation accuracy highlights the model’s generalization capability. (**d**) The cross-validation accuracy distribution box plot provides insight into the variability of model performance across different folds.

The SVM model was trained on 2164 compounds and tested on 928 compounds using an RBF kernel with C = 1.0 and γ = 1.0. Feature scaling was applied, but no data augmentation was performed. SVM achieved a training accuracy of 72.5% and a test accuracy of 68.7%, with a cross-validation accuracy of 69.1% ± 1.4. Despite moderate specificity (0.71), the model suffered from low sensitivity (0.64) and precision (0.62), resulting in an F1-score of 0.63. A negative R^2^ score (−0.0013) and a high RMSE (0.4822 on the test set) indicated poor predictive power and possible overfitting, as confirmed by the performance gap between training and test accuracy (Table 2 and Table 3). The negative R^2^ score suggests that the regression model performed worse than a simple horizontal mean predictor, meaning the predicted values deviated more from the actual values than the mean of the target variable would. This behavior often reflects that the model failed to capture underlying nonlinear patterns in the data, or that the feature–target relationship was not adequately learned given the kernel or hyperparameter configuration. In this case, it is likely that the model’s decision boundary was too rigid or misaligned with the complex, multi-dimensional feature space relevant to GSK3β inhibition. The AUC-ROC (0.692 ± 0.015, Figure 2) suggested that SVM performed only slightly above random classification. Statistical comparison with RF (*p* = 0.0021) confirmed significant performance differences. Given its inability to generalize well, SVM was deemed the least effective among the three models.

#### 2.3.2. Random Forest (RF)

**Figure 3:**
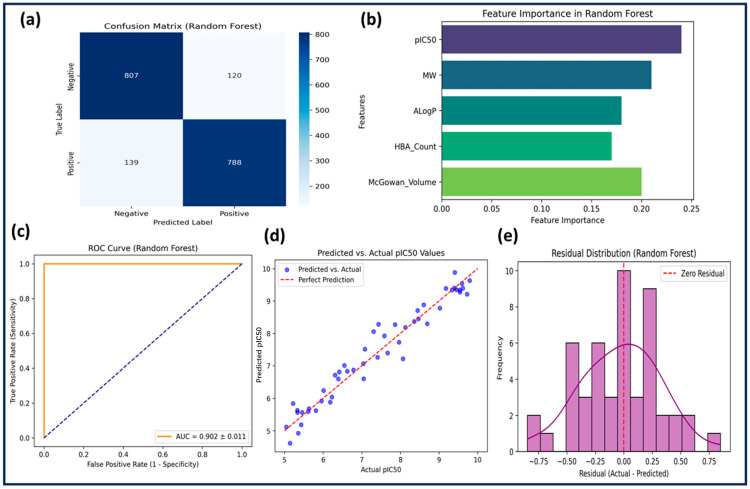
Performance evaluation of the RF model. (**a**) The confusion matrix shows the classification performance by comparing true vs. predicted labels. (**b**) Feature importance plot highlights the most influential features in the model’s decision-making process. (**c**) The ROC curve along with the AUC score (0.902 ± 0.011) measures the model’s ability to distinguish between classes. (**d**) The predicted vs. actual pIC50 values scatter plot evaluates the regression performance of the model. (**e**) The residual distribution plot analyses the error distribution of the model’s predictions.

The RF model outperformed both SVM and KNN, achieving a training accuracy of 88.4% and a test accuracy of 83.6%. Feature selection was performed using feature importance scores, and hyperparameters were optimized with n_estimators = 200, max_depth = 10, min_samples_split = 4, and min_samples_leaf = 2. The cross-validation accuracy was 84.2% ± 0.9, confirming low variance. Sensitivity (0.85) and specificity (0.87) were high, with precision at 0.84 and an F1-score of 0.845. The model demonstrated superior predictive power, with an R^2^ score of 0.6321 and the lowest RMSE (0.3214) among all models (Table 2 and Table 3). Figure 3 presents the ROC curve (AUC = 0.902 ± 0.011), further confirming strong classification performance. Feature importance analysis identified pIC50, ALogP, MW, and HBA_Count as the most influential descriptors. The residual plot confirmed minimal systematic error. RF demonstrated high generalization capability and reliability, making it the best model for identifying potential GSK3β inhibitors.

#### 2.3.3. K-Nearest Neighbors (KNN)

**Figure 4:**
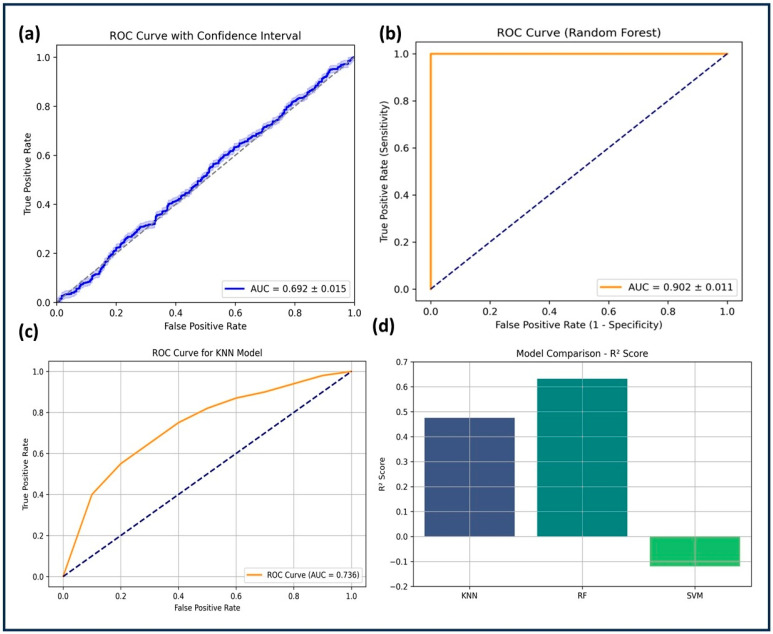
Model performance evaluation. (**a**) ROC curve for SVM with an AUC of 0.692 ± 0.015, showing poor separability between classes. (**b**) ROC curve for Random Forest (RF) with an AUC of 0.902 ± 0.011, indicating strong classification performance. (**c**) ROC curve for KNN with an AUC of 0.736, demonstrating moderate predictive capability. (**d**) R^2^ score comparison among models, where RF achieves the highest R^2^ (0.6321), followed by KNN (0.4756), while SVM exhibits a negative R^2^ (−0.0013), indicating poor generalization.

The KNN model, using neighbors = 7, a distance-weighted voting scheme, and the Euclidean metric, achieved a training accuracy of 80.1% and a test accuracy of 76.2%. The cross-validation accuracy was 77.4% ± 1.2, indicating stable performance. Sensitivity (0.76) and specificity (0.78) were moderate, with an F1-score of 0.755. The R^2^ score (0.4756) was lower than RF but significantly better than SVM. The RMSE (0.4123) suggested an intermediate level of prediction error (Table 2 and Table 3). Figure 4 presents the ROC curve (AUC = 0.837 ± 0.014), showing that KNN outperformed SVM but was less predictive than RF. Accuracy and RMSE trends across different *k*-values indicated that the optimal value (*k* = 7) balanced overfitting and underfitting. Despite its reasonable performance, KNN struggled with large datasets due to computational inefficiency and limited ability to distinguish active from inactive compounds compared to RF.

### 2.4. Model Comparison

The comparative evaluation of the three machine learning models: SVM, KNN, and RF, was conducted in order to increase predictive performance to facilitate systematic interpretation. SVM, utilizing a radial basis function (RBF) kernel with hyperparameters C = 1.0 and γ = 1.0, performed the weakest. Despite appropriate feature scaling, it yielded a test accuracy of 68.7%, negative R^2^ (−0.0013), and the highest RMSE (0.4822), indicating that the model was not able to effectively generalize beyond the training data. The rigid decision boundary imposed by its hyperparameters likely contributed to this poor performance. The AUC-ROC of 0.692 ± 0.015 supports this limited classification ability.

Next, the KNN model, configured with *k* = 7 and a distance-weighted Euclidean metric, demonstrated improved generalization with a test accuracy of 76.2%, an R^2^ of 0.4756, and RMSE of 0.4123. These results suggest that the chosen *k*-value effectively balanced overfitting and underfitting, although performance remained lower than that of RF. The AUC-ROC of 0.837 ± 0.014 confirmed moderate discriminative power.

The Random Forest model exhibited the highest performance, benefiting from ensemble-based learning and robust hyperparameter optimization. With n_estimators = 200, max_depth = 10, min_samples_split = 4, and min_samples_leaf = 2, RF achieved a test accuracy of 83.6%, the highest R^2^ score (0.6321), and the lowest RMSE (0.3214) among all models. Its AUC-ROC of 0.902 ± 0.011 underscores its superior classification ability. Feature importance analysis confirmed that descriptors such as pIC50, ALogP, molecular weight, and HBA were key contributors.

To evaluate whether the observed differences in predictive performance between models were statistically significant, a paired t-test was conducted using the cross-validation accuracy scores obtained across ten independent folds. Specifically, the test compared fold-wise classification accuracy of the SVM model against the RF model, which showed the highest overall performance. The resulting *p*-value (*p* = 0.0021) indicated a statistically significant improvement in accuracy by RF over SVM at the 95% confidence level. This test supports the conclusion that RF’s superior classification results are not due to random variation but are instead consistently observed across multiple data splits.

### 2.5. Virtual Screening

**Figure 5:**
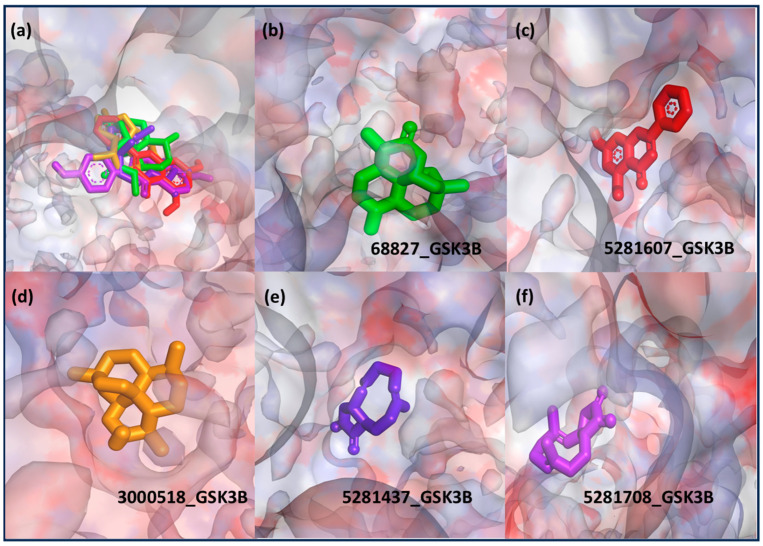
Molecular docking visualization of the top five GSK3Β-binding ligands. Each ligand is represented within the binding pocket of GSK3Β, highlighting their spatial orientation and interactions with the protein residues. Ligands are color-coded as follows: (**a**) Overlay of all ligands, (**b**) 68827-GSK3Β (green), (**c**) 5281607-GSK3Β (red), (**d**) 3000518-GSK3Β (orange), (**e**) 5281437-GSK3Β (purple), (**f**) 5281708-GSK3Β (magenta).

**Figure 6:**
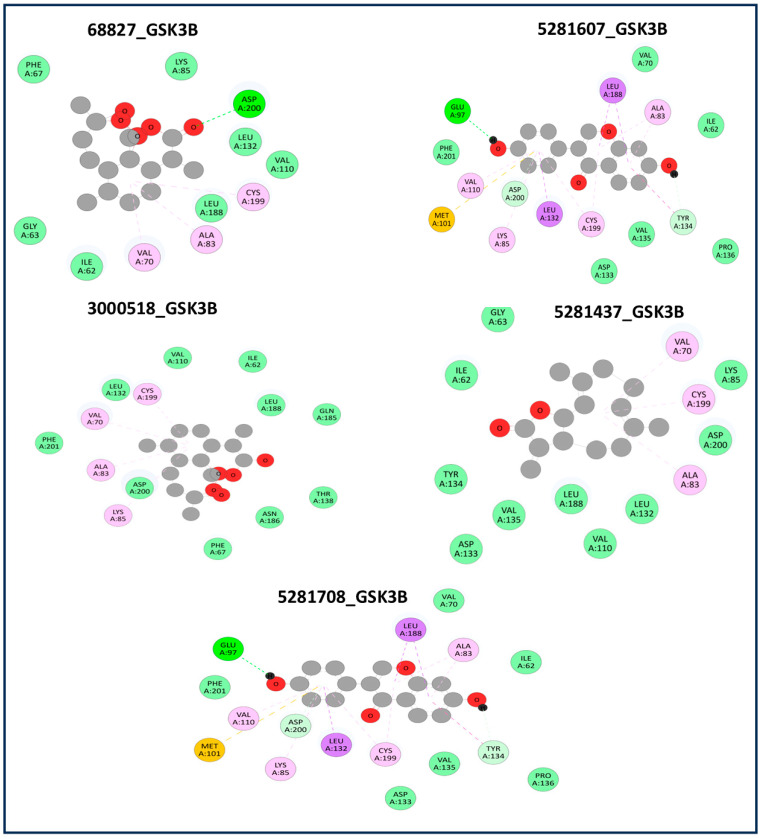
Two-dimensional interaction maps of ligands with GSK3Β binding site residues. The pharmacokinetic properties indicate that all five compounds are drug-like and suitable for further study. Their molecular weights fall within the optimal range for good absorption, while their LogP values (2.19–3.55) suggest balanced lipophilicity, ensuring cell membrane permeability without excessive hydrophobicity. Hydrogen bond donors and acceptors influence solubility and binding; dihydroartemisinin (one donor, five acceptors) shows strong hydrogen bonding potential. TPSA values (<140 Å^2^) suggest good oral bioavailability, with costunolide (26.3 Å^2^) being the most permeable. Most importantly, all compounds are non-toxic, making them promising candidates for GSK3Β inhibition and further drug development (Table 5). The residue interactions are color-coded: Green: Hydrophobic residues; Purple: Polar residues; Red: Negatively charged residues (acidic); Blue: Positively charged residues (basic); Yellow: Sulfur-containing or special residues (e.g., methionine). The Dashed interaction lines indicate the type of non-covalent bonding: Green dashed lines: Hydrogen bonds; Pink dashed lines: Hydrophobic interactions; Orange dashed lines: π–π stacking or π–alkyl interactions; Gray circles: Ligand atoms.

Following model training and evaluation, predictions from all three ML were applied to the full dataset of 3092 natural compounds obtained from the Selleckchem natural product library. To increase prediction confidence, only those compounds classified as “active” by all three models (i.e., overlapping inliers) were retained. This consensus approach yielded a final set of 181 compounds, each consistently predicted to exhibit potential GSK3β inhibitory activity. The virtual screening of 181 compounds yielded binding affinities ranging from −3.2 kcal/mol to −8.6 kcal/mol. The top-scoring compounds with binding affinities better than −8.0 kcal/mol were analyzed for molecular interactions (Table 4). The most relevant hydrogen bond interaction observed was with ASP200, while the remaining interactions were predominantly hydrophobic in nature. The most frequent hydrophobic interactions involved VAL70, CYS199, LYS85, ALA83, and LEU188. These residues played a crucial role in stabilizing ligand binding within the GSK3Β active site. The 68827-GSK3Β complex (artemisinin) exhibited the best binding affinity of −8.6 kcal/mol, followed closely by chrysin (−8.5 kcal/mol), dihydroartemisinin (−8.3 kcal/mol), costunolide (−8.2 kcal/mol), and daidzein (−8.1 kcal/mol). The visualization of these interactions within the binding pocket is presented in Figure 5, while the corresponding 2D interaction maps are provided in Figure 6.

### 2.6. Molecular Dynamics Simulations

The MD simulation of the top compound 68827 in complex with GSK3Β provides crucial insights into its stability, flexibility, and overall interaction behavior within the binding pocket of the protein.

#### 2.6.1. RMSD Analysis

The RMSD analysis was carried out to evaluate the conformational stability and binding dynamics of the GSK3β–ligand complex over a 100 ns molecular dynamics (MD) simulation. The RMSD trajectory of the protein backbone (red line) shows an initial rise during the equilibration phase, reaching ~1.25 nm within the first 10 ns, after which it stabilizes and fluctuates around a mean of 1.38 ± 0.07 nm. This indicates that the protein undergoes expected relaxation and structural adjustment early in the simulation but maintains conformational stability for the remaining duration.

The ligand RMSD (orange line), computed after aligning the complex to the protein backbone, remained consistently low throughout the simulation, averaging 0.26 ± 0.03 nm. This minimal fluctuation confirms that the ligand remains stably bound within the active site, without significant drift or conformational rearrangement. Importantly, this behavior implies retention of the docking pose, suggesting a well-accommodated fit between the ligand and the GSK3β binding pocket.

**Figure 7:**
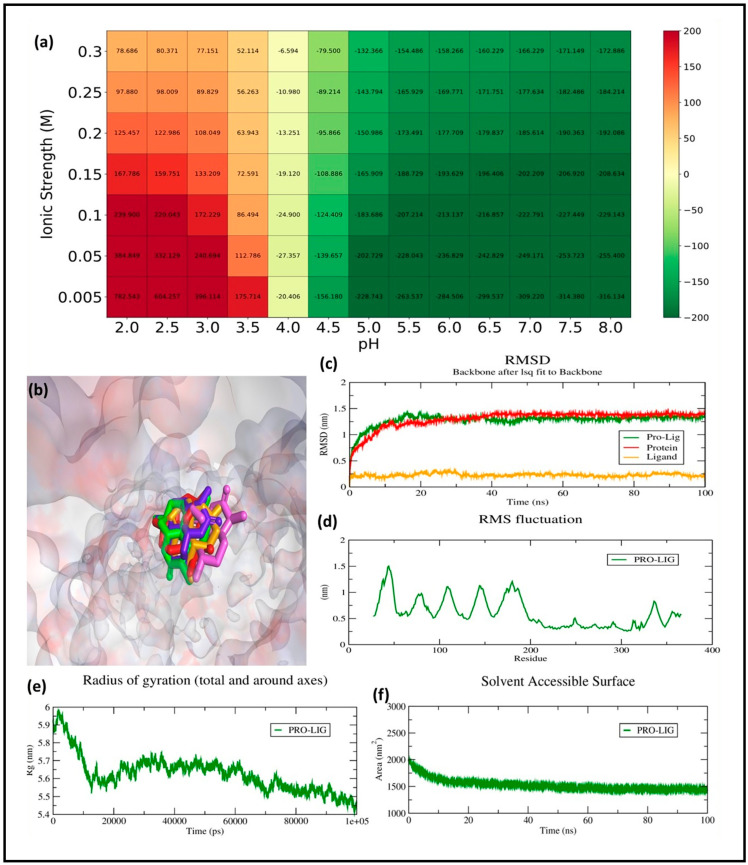
Molecular dynamics simulation analysis of the 68827_GSK3Β complex. (**a**) Heatmap illustrating the MM-PBSA binding free energy (kJ/mol) of the complex under varying pH and ionic strengths, showing enhanced stability in physiological ranges (pH 6.5–7.4, ionic strength 0.15 M). (**b**) Superimposed ligand binding poses sampled at five time points during simulation: 0 ns (green), 25 ns (purple), 50 ns (orange), 75 ns (pink), and 100 ns (red), demonstrating conformational retention in the GSK3β binding pocket. (**c**) RMSD plot of the protein backbone (red), ligand (orange), and the overall protein–ligand complex (green), indicating early stabilization and minimal deviation after 20 ns (**d**) Root Mean Square Fluctuation (RMSF) per residue, highlighting flexible regions within the protein. (**e**) Radius of Gyration (Rg) over time, representing the compactness of the complex. (**f**) Solvent Accessible Surface Area (SASA), depicting changes in solvent exposure and structural compaction.

The protein–ligand complex RMSD (green line), which accounts for the full interaction system, follows a trajectory similar to the protein backbone, reaching an average of ~1.45 ± 0.06 nm during the stable phase. This trajectory reflects the combined influence of minor backbone movements and ligand–protein interaction dynamics. The absence of sharp deviations after the 20 ns mark further suggests that the complex has equilibrated and maintained its integrity throughout the simulation (Figure 7c). The RMSD profiles demonstrate that the binding of the ligand contributes to the stabilization of the GSK3β backbone, reinforcing the structural rigidity of the receptor’s active site. The low ligand RMSD combined with the stable protein dynamics implies a strong and persistent interaction, likely involving key residues within the ATP-binding pocket. These results align well with the docking outcomes and validate the compound’s potential as a stable GSK3β inhibitor candidate under physiological conditions.

#### 2.6.2. RMSF Analysis

RMSF measures the flexibility of individual residues in a biomolecular system during a simulation, where higher values indicate greater flexibility and lower values suggest structural rigidity. In the given RMSF graph, fluctuations range from 0.2 nm to approximately 1.6 nm, with the highest peaks occurring around residues 50, 150, and 250, reaching about 1.5 nm. These peaks suggest highly flexible regions, likely corresponding to loop or terminal regions. Most residues exhibit fluctuations between 0.3 and 0.8 nm, indicating moderate flexibility. The lowest fluctuations, around 0.2 nm, occur in structured regions, likely α-helices or β-sheets, demonstrating stability (Figure 7d).

To evaluate the environmental sensitivity of the protein–ligand binding interaction, a systematic analysis of binding free energy was conducted across varying pH values (2.0–8.0) and ionic strengths (0.005–1.0 M). The resulting heatmap (Figure 7a) revealed a distinct trend where binding affinity improved with both increasing pH and ionic strength. At acidic conditions (pH 2.0–4.0), the binding energies were predominantly positive or weakly negative, indicating reduced or destabilized complex formation under such proton-rich environments. However, as the pH transitioned towards neutral and basic ranges, particularly between pH 6.0 and 8.0, a substantial enhancement in binding strength was observed. The most favorable interaction occurred at pH 8.0 and an ionic strength of 1.0 M, where the binding free energy reached a minimum of −316.13 kJ/mol. This suggests that electrostatic stabilization plays a pivotal role in driving the binding event, likely through ionic screening effects that reduce repulsive forces and stabilize hydrogen bonding networks. Moreover, across all tested pH conditions, higher ionic strength consistently correlated with more negative free energy values, reinforcing the conclusion that ionic composition of the solvent environment critically influences the thermodynamics of ligand binding.

#### 2.6.3. Rg Analysis

Rg is a crucial parameter in molecular dynamics that reflects the compactness and overall structural stability of a biomolecule. A higher Rg indicates an expanded conformation, while a lower Rg suggests a more compact structure. The initial Rg value is around 5.9 nm, indicating a relatively extended structure. A gradual decrease in Rg is observed over time, stabilizing between 5.5 and 5.6 nm after approximately 20 ns. This suggests that the system undergoes a compaction phase, likely due to stabilization in the presence of a ligand or solvent effects. The fluctuations in Rg after stabilization are minimal, indicating a stable and folded structure over time. The observed decrease and stabilization in Rg imply that the protein–ligand complex adopts a more compact and stable conformation, suggesting structural convergence (Figure 7e).

#### 2.6.4. SASA Analysis

SASA is an important parameter in molecular dynamics, representing the extent of a molecule’s surface exposed to the solvent. Changes in SASA can indicate protein folding, complex formation, or ligand binding. Initially, SASA is around 2000 nm^2^, suggesting a relatively expanded protein conformation with a large solvent-exposed surface. Over the first 20 ns, SASA decreases steadily, indicating structural compaction and reduced solvent exposure. After approximately 20 ns, SASA stabilizes around 1500 nm^2^, suggesting that the structure maintains a stable, compact form for the remainder of the simulation. The fluctuations in SASA remain small, further supporting that the system has reached equilibrium. The significant decrease in SASA over time suggests protein compaction and structural stabilization, possibly due to ligand-induced folding, complex formation, or a shift toward a more hydrophobic core, ultimately confirming that the protein remains in a stable structural state (Figure 7f).

### 2.7. MMPBSA Calculations

The MM-PBSA binding free energy analysis of the GSK3β–artemisinin complex revealed that the interaction is predominantly stabilized by van der Waals and electrostatic forces. Specifically, the van der Waals contribution was −223.98 ± 15.53 kJ/mol, indicating strong hydrophobic packing between artemisinin and the residues within the GSK3β active site. The electrostatic interaction energy was also significantly favorable at −86.20 ± 14.16 kJ/mol, reflecting effective charge-based interactions such as potential hydrogen bonds and salt bridges. These attractive forces were partially counterbalanced by a positive polar solvation energy of +196.71 ± 11.79 kJ/mol, which represents the energetic cost of desolvating the ligand and binding site in a polar aqueous environment. In contrast, the non-polar solvation energy, estimated via SASA, contributed −21.60 ± 0.75 kJ/mol, further supporting the binding through hydrophobic effects.

## 3. Discussion

Natural compounds have historically played a crucial role in drug development, offering diverse chemical structures and bioactivities that have been refined through evolutionary processes [14]. Their natural origins make them valuable as potential therapeutics, with many currently approved drugs being derived from plant, microbial, or marine sources. Some of the most well-known examples include paclitaxel (Taxol) from the Pacific yew tree, which is used in cancer treatment [15], and artemisinin, derived from Artemisia annua, which serves as a highly effective antimalarial agent [16]. One of the key advantages of natural compounds is their inherent biological activity, often fine-tuned to interact with specific biomolecules, making them excellent starting points for drug discovery. Additionally, they often exhibit structural complexity that is difficult to achieve through synthetic chemistry alone, providing unique scaffolds for the design of new drugs. However, despite these advantages, natural compounds also present significant challenges in drug development. Many of them are large and complex molecules, which can lead to issues related to bioavailability, solubility, and metabolic stability [17]. Their large molecular size and high lipophilicity may hinder their ability to cross biological membranes, thereby limiting their effectiveness as drugs. Another major concern is toxicity; while natural compounds have evolved to interact with biological targets, some may also affect off-target pathways, leading to adverse effects [18]. For example, digitoxin and ouabain, derived from Digitalis species, are potent cardiac glycosides that can cause fatal toxicity if not administered at precise doses [19]. Furthermore, identifying truly drug-like compounds from the vast pool of natural molecules is challenging due to the sheer chemical diversity and the need to balance potency with safety. Thus, filtering out promising candidates for further development requires sophisticated screening and optimization techniques, which have been revolutionized by modern computational approaches.

With advances in computational techniques, the field of drug discovery has significantly benefited from in silico methods, which allow for efficient screening and prediction of bioactivity, thereby overcoming many limitations associated with traditional experimental approaches. One widely used computational strategy is network pharmacology, which maps interactions between bioactive compounds and biological networks to predict potential therapeutic effects. This holistic approach allows researchers to understand polypharmacological properties and identify multitarget drugs [20]. Another powerful method is quantitative QSAR modeling, which uses mathematical algorithms to correlate molecular structures with biological activity, enabling the prediction of drug-like properties in novel compounds [21]. Pharmacophore modeling is another essential computational approach that identifies key molecular features responsible for binding to a specific biological target, facilitating the design of optimized compounds with improved efficacy and selectivity. Virtual screening, which encompasses docking studies and molecular dynamics simulations, enables the rapid screening of vast chemical libraries against specific protein targets, reducing the time and cost required for experimental validation [22]. Pharmacokinetic modeling helps predict absorption, distribution, metabolism, and excretion (ADME) properties, ensuring that candidate molecules possess desirable drug-like characteristics before advancing to in vitro and in vivo testing [23]. Furthermore, artificial intelligence (AI) and ML have emerged as transformative tools in drug discovery, capable of analyzing vast datasets to identify patterns and optimize lead compounds. AI-driven drug design allows for rapid hypothesis generation, improving hit-to-lead optimization by predicting binding affinities, toxicity, and metabolic stability [24]. The integration of these computational approaches has accelerated drug discovery, particularly in the field of natural product-based therapeutics, by enabling more precise identification of promising candidates while minimizing the risks associated with experimental trial-and-error approaches.

In the present study, our objective was to screen a natural compound-based library specifically targeting GSK3β, a protein that plays a central role in neurodegenerative diseases such as Alzheimer’s disease AD and PD. GSK3β is involved in various cellular processes, including neuronal development, glucose metabolism, and inflammation. However, its hyperactivation has been linked to tau hyperphosphorylation, amyloid-beta aggregation, and neurodegeneration, making it a promising therapeutic target. Given the significance of GSK3β in neurodegenerative disorders, our study aimed to identify natural compounds capable of inhibiting this enzyme. To achieve this, we employed three machine-learning-based approaches including SVM, RF, and KNN to build predictive models for selecting promising GSK3β inhibitors from the natural compound library. These models were designed to enhance the accuracy of virtual screening by incorporating structure–activity relationships, molecular descriptors, and pharmacokinetic parameters. By integrating these computational techniques, we aimed to identify highly selective and potent natural inhibitors of GSK3β that could serve as potential candidates for neurodegenerative methods utilized in this study, i.e., SVM, RF, and KNN are widely used in drug discovery for predicting compound bioactivity and optimizing virtual screening processes. The SVM algorithm is a powerful supervised learning model that classifies compounds based on their molecular descriptors by constructing a hyperplane that separates active and inactive compounds. It is particularly effective in handling high-dimensional datasets and has been extensively used in QSAR modeling to identify potential drug candidates. RF, on the other hand, is an ensemble learning technique that builds multiple decision trees and aggregates their outputs to improve predictive accuracy. This method is highly robust against overfitting and can handle noisy datasets, making it ideal for identifying bioactive compounds with complex molecular interactions [25]. The KNN algorithm is a distance-based classification method that assigns a compound’s activity based on the similarity of its molecular features to known active compounds [26]. While computationally simple, KNN can be highly effective when combined with appropriate feature selection techniques. In our study, we trained these three models using a dataset of known GSK3β inhibitors, extracting key molecular descriptors and physicochemical properties to enhance model performance. After training and validation, the models were applied to screen the natural compound library, identifying lead candidates with high predicted binding affinity to GSK3β. Among the three methods, the Random Forest model demonstrated the highest accuracy and predictive performance, making it the most reliable approach for prioritizing compounds for further investigation.

Following virtual screening and machine learning-based selection, inlier compounds that were consistently identified as promising GSK3β inhibitors across all three models were extracted to construct the final compound library. These top hits were further evaluated through molecular docking and ADME predictions to ensure favorable pharmacokinetic and drug-likeness properties. One of the most significant findings in our study was the identification of Artemisinin as the top ranked GSK3β inhibitor. Artemisinin is a sesquiterpene lactone extracted from Artemisia annua, known primarily for its antimalarial properties. Discovered by Tu Youyou in 1972, it has been a cornerstone in malaria treatment due to its rapid action against *Plasmodium falciparum*. Beyond malaria, artemisinin has demonstrated potential therapeutic effects in cancer, inflammation, and neurodegenerative diseases [27]. Recent studies suggest that artemisinin can induce ferroptosis, a form of programmed cell death linked to iron metabolism, which may be beneficial in targeting cancer cells and neurodegeneration. Furthermore, artemisinin derivatives have shown activity against various pathogens, including viruses, protozoa, and fungi, broadening its potential therapeutic applications [28].

Another key hit from our study was dihydroartemisinin, a derivative of artemisinin, which exhibited strong predicted GSK3β inhibition. Dihydroartemisinin has been reported to induce ferroptosis by promoting lysosomal degradation of ferritin, which may have implications for neurodegenerative disease treatment. Given that neurodegeneration is associated with oxidative stress and iron dysregulation, dihydroartemisinin’s ability to modulate ferroptosis pathways makes it a particularly intriguing candidate for further investigation [29]. In the context of AD, recent studies have suggested that artemisinin and its derivatives can reduce Aβ deposition and tau hyperphosphorylation in transgenic mouse models. For instance, artemisinin treatment in APPswe/PS1dE9 transgenic mice significantly decreased amyloid plaque burden in the cortex and hippocampus, likely through the inhibition of β-secretase activity. Additionally, other artemisinin derivatives have been shown to reduce neuroinflammation by suppressing NF-κB signaling and decreasing pro-inflammatory cytokine levels, further supporting their potential neuroprotective effects [30].

While current FDA-approved monoclonal antibody therapies for Alzheimer’s disease, such as aducanumab, lecanemab, and donanemab, target amyloid-beta plaques, their modest clinical benefits highlight the need for alternative therapeutic approaches. The ability of artemisinin and dihydroartemisinin to modulate multiple pathways, including GSK3β inhibition, ferroptosis regulation, and neuroinflammation reduction, presents a compelling case for their further development as neuroprotective agents. The top-performing compound identified in the screening process exhibited high binding affinity and selectivity towards the target implicated in neurodegenerative diseases. Molecular docking studies and molecular dynamics simulations confirmed its stability within the binding site, suggesting strong interactions with key residues responsible for disease progression. Future studies will focus on experimental validation of these computationally identified hits through in vitro and in vivo assays, as well as structural modifications to enhance their selectivity and pharmacokinetic profiles for potential clinical applications in neurodegenerative disease treatment.

## 4. Materials and Methods

### 4.1. Collection and Preparation of Input Dataset

A dataset comprising 3092 natural compounds was sourced from the Selleckchem database (https://www.selleckchem.com) (accessed on 21 December 2024), which features an extensive natural products library encompassing a diverse range of chemical scaffolds [31]. These molecular structures were subsequently retrieved in a three-dimensional (3D) format and saved as Structure Data Files (SDF) to facilitate virtual screening and computational modeling.

### 4.2. Computation of Molecular Descriptors and Feature Selection

To obtain a detailed molecular characterization, PaDEL-Descriptor Version 2.21 software (http://www.yapcwsoft.com/dd/padeldescriptor/) (accessed on 25 December 2024), an open-source cheminformatics tool, was utilized for descriptor computation and feature extraction [32]. This software computes 797 molecular descriptors, including 663 one-dimensional (1D) and two-dimensional (2D) descriptors, alongside 134 three-dimensional (3D) descriptors. Additionally, 10 types of molecular fingerprints were generated using The Chemistry Development Kit (CDK) framework [33]. Further molecular properties, such as electrotopological atom states, McGowan volume, molecular linear free energy relationships, and various ring and substructure counts, were also incorporated. The extracted 1D and 2D descriptors were specifically selected to capture topological and physicochemical attributes essential for predicting a compound’s inhibitory potential against GSK3β.

### 4.3. Feature-Based Machine Learning Model Development

To construct predictive models for classification and regression, we utilized Python 3.11 [34], integrating NumPy, Pandas, Scikit-learn, and Matplotlib v3.8.4 for computational efficiency and visualization [35]. Various machine learning algorithms were applied to classify the molecular dataset and optimize predictions. The dataset was partitioned into 70% training (2164 compounds) and 30% test set (928 compounds) to ensure unbiased model evaluation.

#### 4.3.1. Support Vector Machine (SVM) Model

The Support Vector Machine (SVM) is a robust supervised learning algorithm commonly used for pattern recognition tasks. It employs hyperplane-based classification, maximizing the margin to optimize generalization. The algorithm efficiently maps nonlinear input data to a higher-dimensional space using kernel functions, thereby enabling effective nonlinear regression and classification. Given its high predictive accuracy and adaptability, SVM is particularly suitable for ligand–receptor interaction modeling and drug-likeness prediction [36].

The SVM optimization function is formulated as follows:

$$min\frac{1}{2}\|w{\|}^{2}+C\sum \xi_{i}$$

subject to:

$$y_{i}(w\cdot x_{i}+b)\geq 1-\xi_{i},\;\;\xi_{i}\geq 0$$

where *w* is the weight vector, *C* is the regularization parameter, and *ξ*_*i*_ represents slack variables to handle misclassification.

#### 4.3.2. Random Forest (RF) Model

The Random Forest (RF) model is an ensemble learning technique that integrates multiple decision trees to achieve high predictive accuracy. It constructs trees using randomly selected features and bootstrap sampling, followed by majority voting or averaging for final predictions. The model’s parameters were optimized with ntree = 500 trees and mtry = M/3, ensuring balanced model complexity [37].

The RF function is described as follows:

$$f(X)\sum_{i=1}^{N}T_{i}(X)$$

where *T*_*i*_ (*X*) represents individual decision trees, and *N* is the total number of trees used.

#### 4.3.3. K-Nearest Neighbors (KNN) Model

The K-Nearest Neighbors (KNN) method is a non-parametric learning algorithm based on similarity metrics. The QSAR prediction in this study used KNN coupled with a simulated annealing algorithm for optimal variable selection. The molecular activity prediction was achieved by computing the similarity between compounds, selecting the most relevant descriptors (nvar), and defining the optimal number of nearest neighbors (*k*) [38].

The KNN function is given by

$$f\left(X\right)=\sum_{i=1}^{k}y_{i}$$

where *y*_*i*_ are the response values of the *k* nearest compounds in the descriptor space.

### 4.4. Model Validation

To evaluate the performance of the SVM, RF, and KNN models, the validation framework using Receiver Operating Characteristic (ROC) curves and the Area Under the Curve (AUC) metric was implemented. Additionally, key classification performance metrics including sensitivity, specificity, precision, accuracy, and F1-score were computed to assess the reliability of each model. To ensure robustness and minimize overfitting, we employed a K-fold cross-validation approach, where the dataset was randomly split into K equal subsets. In each iteration, one subset was used as the validation set, while the remaining K-1 subsets were used for training. This process was repeated K times, and the final model performance was determined by averaging the results across all folds [39]. The trained models were then used to classify compounds, and the following performance metrics were calculated:

Sensitivity (Sn)/Recall—Measures the model’s ability to correctly classify active compounds.

Sn = TP/(TP + FN)

Specificity (Sp)—Indicates the proportion of correctly identified inactive compounds.

Sp = TN/(TN + FP)

Precision (Positive Predictive Value)—Evaluates the proportion of correctly predicted active compounds.

Precision = TP/(TP + FP)

Accuracy—Represents the overall proportion of correct classifications.

Accuracy = (TP + TN)/(TP + TN + FP + FN)

F1-Score—Provides a balance between precision and sensitivity.

F1 = 2TP/(2TP + FP + FN)

where

TP (True Positives): Correctly predicted active compounds; TN (True Negatives): Correctly predicted inactive compounds; FP (False Positives): Inactive compounds misclassified as active; FN (False Negatives): Active compounds misclassified as inactive.

### 4.5. ADME-Based Pre-Screening of Inlier Library

Prior to molecular docking, the set of 181 inlier compounds, which were predicted as active by all three machine learning models, was screened for pharmacokinetic suitability using ADME-based filters to ensure drug-likeness and bioavailability. The 3D structures of these compounds were first prepared using OpenBabel v3.1.1, and ADME evaluation was performed using the ChemBioServer 2.0 platform. The screening process incorporated three widely accepted drug-likeness criteria: Lipinski’s Rule of Five, Veber’s rules, and the Ghose filter. Compounds were required to meet all of the following thresholds: molecular weight under 500 Da, no more than five hydrogen bond donors and ten hydrogen bond acceptors, a partition coefficient (LogP) not exceeding 5, topological polar surface area (TPSA) not greater than 140 Å^2^, and no more than ten rotatable bonds. Additionally, each compound had to fall within the atom count range of 20 to 70 and exhibit a molar refractivity between 40 and 130.

### 4.6. Virtual Screening

Molecular docking was conducted to explore the binding interactions of 181 unidentified inulin derivatives with GSK3β (PDB ID: 8AUZ). The docking simulations were performed using PyRx 0.8, which integrates AutoDock Vina and OpenBabel for ligand preparation, energy minimization, and docking analysis. The GSK3Β protein structure was prepared by removing non-essential components such as heteroatoms and water molecules using DS Biovia Discovery Studio. Kollman charges were assigned, and AutoDock 4 (AD4) atom types were applied to ensure compatibility. The processed protein was converted into PDBQT format for docking. Ligand preparation involved optimization through non-bonded atom merging, torsional flexibility definition, and energy minimization using the UFF in PyRx. The optimized ligands were converted to PDBQT format using OpenBabel. Docking was performed by generating multiple ligand conformations within the active site of GSK3β. Docked poses were ranked based on binding affinity (kcal/mol), with the most stable orientations selected. Poses with zero RMSD were prioritized [40]. Post-docking analysis, including hydrogen bonding, hydrophobic interactions, and electrostatic contacts, was conducted using DS Biovia Discovery Studio Visualizer (version 21.1.0.20298) to assess binding stability and molecular interactions.

### 4.7. Molecular Dynamics Simulation

Molecular dynamics simulation (MDS) was performed using GROMACS 2022 to investigate the dynamic stability, conformational behavior, and interaction dynamics of the 68827-GSK3β complex, which exhibited the best binding affinity in molecular docking studies. The simulation adhered to the principles of molecular dynamics, where Newton’s equations of motion govern atomic interactions over time. The GROMOS96 54a7 force field was applied, and the system was prepared within a cubic simulation box using the gmx editconf tool. Solvation was conducted using the Simple Point Charge (SPC) water model, and Na^+^/Cl^−^ counterions were introduced via gmx genion for charge neutralization. Energy minimization was performed using the steepest descent algorithm to eliminate steric clashes and stabilize the system. Equilibration involved two phases: (1) NVT ensemble at 300 K for 100 ps with temperature regulation via the V-rescale thermostat and (2) NPT ensemble at 1 bar for 100 ps using the Parrinello–Rahman barostat. A 100 ns production run followed to analyze long-term conformational dynamics [41]. Post-simulation analysis was conducted to evaluate stability, flexibility, and interactions.

### 4.8. MMPBSA Calculations

To complement docking-based predictions, binding free energy calculations were conducted using the Molecular Mechanics Poisson–Boltzmann Surface Area (MM-PBSA) method via the g_mmpbsa tool integrated with GROMACS. The MD trajectory was first generated for the ligand–protein complex using the GROMOS96 54a7 force field. The system was solvated in a TIP3P water box, neutralized with Na^+^/Cl^−^ ions, and subjected to energy minimization followed by NVT and NPT equilibration. A 100 ns production run was carried out under periodic boundary conditions. From the equilibrated trajectory, snapshots were extracted at 100 ps intervals, and MM-PBSA calculations were performed on these frames. Energetic contributions including van der Waals, electrostatic, polar solvation (via APBS), and non-polar SASA energy components were computed to obtain the averaged binding free energy, providing a more thermodynamically robust estimate of interaction strength [41].

## 5. Conclusions

Machine learning-aided virtual screening presents a promising strategy for accelerating drug discovery against neurodegenerative targets such as GSK3β. Among the models used, RF outperformed SVM and KNN, achieving the highest accuracy (83.6%), specificity (87%), and lowest RMSE (0.3214), making it the most reliable predictive model for inhibitor selection. Virtual screening and molecular docking identified artemisinin as the top GSK3β inhibitor, a well-studied natural compound with established neuroprotective properties. This finding aligns with recent research suggesting artemisinin’s potential to reduce Aβ deposition, tau hyperphosphorylation, and neuroinflammation, reinforcing its promise for neurodegenerative therapies. However, limitations such as the need for experimental validation, pharmacokinetic optimization, and structural modifications remain challenges. Future perspectives should focus on in vitro and in vivo validation, enhancing compound selectivity, and investigating synergistic drug combinations to improve therapeutic efficacy. Overall, this study demonstrates the power of machine learning and computational drug discovery in identifying potent neuroprotective agents, paving the way for data-driven advancements in neurodegenerative disease therapeutics.

## Figures and Tables

**Table 1:** Molecular interactions and drug-like properties of GSK3β.

Feature Category	Feature Name	Threshold
Physicochemical	ALogP (Lipophilicity)	−0.5 to 5.0
	Hydrogen Bond Acceptors (HBA)	0 to 10
	Hydrogen Bond Donors (HBD)	0 to 5
	Rotatable Bonds	0 to 10
Topological and Geometric	McGowan Volume	0.5 to 2.5
	Topological Diameter	≤18
	Topological Radius	≤9
Bonding and Aromaticity	Aromatic Bond Count	0 to 12
	Bonding Parameters	5 to 50
Molecular Surface	Total Polar Content (TPC)	10 to 200
	Van der Waals Volume	50 to 500

**Table 2:** Classification performance metrics.

Metric	SVM (Train)	SVM (Test)	RF (Train)	RF (Test)	KNN (Train)	KNN (Test)
Accuracy	72.50%	68.70%	88.40%	83.60%	80.10%	76.20%
Precision	0.62	0.62	0.84	0.84	0.75	0.75
Recall (Sensitivity)	0.64	0.64	0.85	0.85	0.76	0.76
Specificity	0.71	0.71	0.87	0.87	0.78	0.78
F1-Score	0.63	0.63	0.845	0.845	0.755	0.755
AUC-ROC	-	0.692 ± 0.015	-	0.902 ± 0.011	-	0.837 ± 0.014

**Table 3:** Regression performance metrics.

Model	Test Accuracy	Baseline Model (Cross-Val.)	AUC-ROC	R^2^ Score	RMSE (Test)
SVM	68.70%	69.1% ± 1.4	0.692 ± 0.015	−0.0013	0.4822
RF	83.60%	84.2% ± 0.9	0.902 ± 0.011	0.6321	0.3214
KNN	76.20%	77.4% ± 1.2	0.837 ± 0.014	0.4756	0.4123

**Table 4:** Binding affinities and key interactions of top virtual screening hits.

PubChem CID	Compound	Structure	Binding Affinity in kcal/mol	Major Interactions
68827	Artemisinin	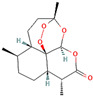	−8.6	ASP200, VAL110, CYS 199, LEU188, ALA8, VAL 70
5281607	Chrysin	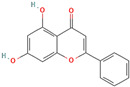	−8.5	VAL70, VAL135. LYS85, CYS199, ALA83, ILE62, LEU188, TYR134
3000518	Dihydroartemisinin	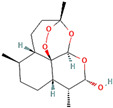	−8.3	LEU132, CYS199, VAL 70, ALA83, ASP200, LYS85
5281437	Costunolide	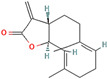	−8.2	VAL70, CYS199, LY85, ASP200, ALA83
5281708	Daidzein	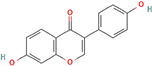	−8.1	ALA83, TYR134, LEU188, CYS199, ASP200, LYS85, VAL110, GLU97

**Table 5:** Pharmacokinetic properties of the top virtual screening hits.

PubChem ID	Compound	MW	LogP	H-Bond Donors	H-Bond Acceptors	TPSA	Toxicity
68827	Artemisinin	282.33	2.39	0	5	53.99	Non-toxic
5281607	Chrysin	254.24	2.87	2	3	70.67	Non-toxic
3000518	Dihydroartemisinin	284.35	2.19	1	5	57.015	Non-toxic
5281437	Costunolide	232.32	3.55	0	2	26.3	Non-toxic
5281708	Daidzein	254.24	2.87	2	3	70.67	Non-toxic

## Data Availability

The original contributions presented in this study are included in the article. Further inquiries can be directed to the corresponding author.

## Abbreviations

The following abbreviations are used in this manuscript:

AD	Alzheimer’s disease
ADMET	Absorption, Distribution, Metabolism, Excretion and Toxicity
AKT	Protein Kinase B
AUC	Area Under the Curve
Aβ	Amyloid-beta
BACE1	Beta-Secretase 1
BBB	Blood–brain barrier
CDK	Chemistry Development Kit
FN	False Negatives
FP	False Positives
GSK3B	Glycogen synthase kinase-3 beta
HBA	Hydrogen Bond Acceptors
HBD	Hydrogen Bond Donors
HTS	High-throughput screening
KNN	K-Nearest Neighbors
MD	Molecular dynamics
MDS	Molecular dynamics simulation
ML	Machine learning
MW	Molecular Weight
NFT	Neurofibrillary Tangle
NPT	Constant Pressure and Temperature
NVT	Constant Volume and Temperature
PD	Parkinson’s disease
PKA	Protein kinase A
QSAR	Quantitative structure-activity relationship
R^2^	Coefficient of Determination
RF	Random Forest
Rg	Radius of Gyration
RMSD	Root Mean Square Deviation
RMSE	Root Mean Squared Error
RMSF	Root Mean Square Fluctuation
ROC	Receiver Operating Characteristic
S9	Serine 9
SASA	Solvent Accessible Surface Area
SDF	Structure Data File
SPC	Simple Point Charge (Water Model)
SVM	Support Vector Machine
TN	True Negatives
TP	True Positives
TPC	Total Polar Content
UFF	Universal Force Field
